# Preparing to caress: a neural signature of social bonding

**DOI:** 10.3389/fpsyg.2015.00016

**Published:** 2015-01-28

**Authors:** Rafaela R. Campagnoli, Laura Krutman, Claudia D. Vargas, Isabela Lobo, Jose M. Oliveira, Leticia Oliveira, Mirtes G. Pereira, Isabel A. David, Eliane Volchan

**Affiliations:** ^1^Laboratory of Neurobiology, Institute of Biophysics Carlos Chagas Filho, Universidade Federal do Rio de JaneiroRio de Janeiro, Brazil; ^2^Laboratory of Behavioral Neurophysiology, Biomedical Institute, Universidade Federal FluminenseNiterói, Brazil

**Keywords:** social bonding, caress, affiliative behavior, readiness potential, EEG/ERP, motor planning, active touch, grooming

## Abstract

It is assumed that social bonds in humans have consequences for virtually all aspects of behavior. Social touch-based contact, particularly hand caressing, plays an important role in social bonding. Pre-programmed neural circuits likely support actions (or predispositions to act) toward caressing contacts. We searched for pre-set motor substrates toward caressing by exposing volunteers to bonding cues and having them gently stroke a very soft cloth, a caress-like movement. The bonding cues were pictures with interacting dyads and the control pictures presented non-interacting dyads. We focused on the readiness potential, an electroencephalographic marker of motor preparation that precedes movement execution. The amplitude of the readiness potential preceding the grasping of pleasant emotional-laden stimuli was previously shown to be reduced compared with neutral ones. Fingers flexor electromyography measured action output. The rationale here is that stroking the soft cloth when previously exposed to bonding cues, a compatible context, would result in smaller amplitudes of readiness potentials, as compared to the context with no such cues. Exposure to the bonding pictures increased subjective feelings of sociability and decreased feelings of isolation. Participants who more frequently engage in mutual caress/groom a “significant other” in daily life initiated the motor preparation earlier, reinforcing the caress-like nature of the task. As hypothesized, readiness potentials preceding the caressing of the soft cloth were significantly reduced under exposure to bonding as compared to control pictures. Furthermore, an increased fingers flexor electromyographic activity was identified under exposure to the former as compared to the latter pictures. The facilitatory effects are likely due to the recruitment of pre-set cortical motor repertoires related to caress-like movements, emphasizing the distinctiveness of neural signatures for caress-like movements.

“…*human hands are much more than instruments for manipulation – indeed, they are caressing organs. The fingers of the human hand can be extended fully as well as delicately flexed, allowing the hand as a totality to accommodate to any curved surface of the body in a caressing touch, more or less in the same manner that the tongue of other animals does. In modern human beings, hand caresses occupy the whole hand with the fingers flexing adequately to fit the caressed surface in a gentle holding touch*.”

(Maturana and Verden-Zoller, 2008)

## Introduction

In social mammals, including humans, it has been proposed that predisposition to actions of parental care, as well as to actions of formation and maintenance of social bonds, constitutes an essential feature for preserving survival. Body nearness and interpersonal social touch is a prominent component of social bonding in humans (Carter et al., 2005; Nelson and Geher, 2007; Dunbar, 2010; Gallace and Spence, 2010). Caressing and seeking caresses are essential motivational drives. Studies have addressed the neural underpinnings of receiving pleasant touch, from peripheral receptors in the skin to the brain (Löken et al., 2009; Morrison et al., 2010; Gazzola et al., 2012; Lloyd et al., 2013; Ackerley et al., 2014). Equally important but less explored is the investigation of the action of caressing (Ebisch et al., 2014). Given the adaptive relevance of interpersonal social touch, the existence of pre-set motor neural circuits associated with caress actions is very likely.

Preset motor repertoires for ethologically relevant actions in the monkey cortex were shown by Graziano (2006) through mapping studies with microstimulation of motor cortical areas In that study, behaviorally useful actions included withdrawal movements, such as defensive maneuvers to protect the body surface, and approach movements, such as reaching, grasping, and manipulation motions. Ethologically relevant hand/mouth representations were shown recently in the human precentral gyrus, through intra-cortical stimulation and recordings in peroperative settings (Desmurget et al., 2014). A pioneer study (Oliveira et al., 2012) brought evidence in humans for preset representations in the motor cortex associated with grasping pleasant objects. The authors worked with the readiness potential (*Bereitschaftspotential*), a slow negative electroencephalographic activity preceding a self-initiated movement (Deecke et al., 1969) which reflects motor preparation involving the supplementary, premotor and primary motor areas (Ikeda et al., 1992; Yazawa et al., 2000; Shibasaki and Hallett, 2006). The work by Oliveira et al. (2012) analyzed the amplitude of the readiness potential preceding the interaction with emotional-laden stimuli presented in transparent cylinders, balanced in weight between the emotional categories. For each trial, upon stimulus presentation, participants waited a few seconds and grasped the cylinder and brought it close to one's body. Compared with neutral stimuli, the grasping of pleasant stimuli was preceded by a readiness potential of lower amplitude. On the other hand, grasping and bringing unpleasant stimuli close to one's body was associated with larger readiness potentials compared to neutral and pleasant stimuli. Smaller readiness potential amplitudes found for pleasant stimuli could imply the recruitment of pre-set motor repertoires directed to a compatible movement, that is, approach pleasant items; whereas higher amplitudes found for unpleasant stimuli would emerge from an incompatibility between the required action (approach) and the preset networks to repel the unpleasant stimuli. That is, approaching unpleasant stimuli would mobilize more resources to comply with the instructions, while approaching pleasant stimuli would be preset and easier to recruit. This is in line with evidence from slow wave potentials studies showing that higher amplitudes are associated with movement complexity (Shibasaki and Hallett, 2006) and possibly more sensory-motor resource mobilization (McCallum, 1993).

Interestingly and of relevance to the present work, Graziano's (2006) work showed that stimulation sites over a large region of the monkey motor cortex caused the hand to move into a restricted region of central space and the fingers to shape in a specific manner including an apparent precision grip (thumb against forefinger) or a power grip (fist), bearing resemblance with the movements when grooming the fur of another monkey. In humans, Souza et al. (2012) conducted a behavioral study that suggested an imprinted predisposition to grooming-like movement. The authors showed that exposure to affiliative pictures facilitated a simple detection task, e.g., yielded faster reaction times, if performed with index finger flexion (compatible with grooming); but imposed costs to the task, e.g., yielded slower reaction times, if performed with finger extension (incompatible with grooming).

Infants of 18 months old are already able to perceive subtle bonding cues in the environment and engage in connected behavior (Over and Carpenter, 2009). Adult observers can extract bonding information from minimalistic moving point-light displays depicting two interacting individuals (Centelles et al., 2011). In that study, among other regions, the premotor and supplementary motor cortices were modulated during the observation of interacting scenes. Further, modulation of motor areas was reported by Caria et al. (2012) when participants passively observed pictures of babies' faces. This was interpreted as reflecting an implicit preparation to interact with babies. In a posturographic study, exposure to affiliative pictures induced significant motor modulation in the observer, interpreted as a tendency to favor social bonds (Facchinetti et al., 2006).

To provide evidence of imprinted dispositions to caress in the human brain, we designed a task resembling a caress-like movement and probed the readiness potential, as a brain marker of motor preparation for this movement. We investigate the readiness potential that precedes the stroking of a soft cloth under exposure to pictures depicting either scenes of socially interacting dyads or scenes of non-interacting dyads. The rationale is that stroking the soft cloth when previously exposed to bonding cues, would recruit pre-set cortical motor repertoires related to caress-like movements. Under no such cues, the motor preparation would be much less tuned, or not tuned at all, to caressing circuits. Based on the study of Oliveira et al. (2012), we expected smaller amplitudes of readiness potentials in the presence of bonding cues (interacting dyads), a compatible context, as compared to the context with no such cues.

## Methods

### Ethics statement

This study was approved by the local institutional Ethics Review Board. All participants provided informed consent prior to the assessment.

### Participants

A total of 21 right-handed students (11 women, *M* = 22.6 years old; *SD* = 2.84) participated in this study. None reported any psychiatric or neurological disease or use of any medication acting on the central nervous system. Handedness was assessed using the Edinburgh inventory (Oldfield, 1971). The participants were naive with respect to the purpose of the experiment.

### Visual stimuli

All pictures displayed two people. The dyads were either an adult and a child or two children presenting cues of kinship. Some of the pictures used were purchased from Getty Images^®^ (www.gettyimages.com), and others were provided by members of the laboratory. Two stimulus conditions (30 pictures each) were selected: with and without social interaction. For the “bonding” condition; the dyad was embracing each other, and/or kissing, and/or engaging in eye contact. For the “control” condition, the individuals were directing their gaze, face, and body anywhere except toward each other. Examples of each condition are presented in Supplementary Material.

### Electrophysiological recordings

Surface electromyographic (EMG) activity was recorded from the flexor digitorum superficialis muscle of the left arm, a flexor muscle of the fingers, as a marker of the initiation of fingers movements. EMG activity was also recorded from the extensor digitorum muscle of the left arm as a control. Four Ag/AgCl electrodes (diameter: 8 mm; inter-electrode distance: 2 cm) connected to an MP150 amplifier (BIOPAC Systems Inc.) were used. A reference electrode was affixed to the left lateral epicondylus. The EMG signal was acquired at a sampling rate of 1000 Hz with a gain of 1000 and analogically filtered online (band-pass: 10–500 Hz).

The electroencephalographic (EEG) signal was recorded from 23 plumb electrodes (EMSAMED, Rio de Janeiro, BRAZIL) placed according to the international 10–20 system. All electrodes were referenced to channel Cz during the recording session and re-referenced to the averaged mastoids. The electrodes were affixed using conductive paste. Impedances were maintained below 5 kΩ. The EEG signal was sampled at 400 Hz, and data were filtered during acquisition using a 0.1-Hz high-pass filter.

### Self-reported mood: hope for closeness and fear of rejection

A list of 27 adjectives derived from the work of Wirth and Schultheiss (2006) were presented in random order. Participants were instructed to report how much each adjective reflected their present mood out of four options (definitely not, slightly not, slightly, or definitely). Unsuspected by the participants, the list contained two measures of moods, (a) “hope for closeness” (sociable, affectionate, gregarious, warm, loving, compassionate—positively keyed items; cold, aloof, distant, remote, detached, unfriendly, independent—negatively keyed items); and (b) “fear of rejection” (lost, forlorn, isolated, abandoned, panicky, lonely, rejected—positively keyed items; safe, secure, protected, accepted, attached, loved, trusting—negatively keyed items). This list was applied twice during the experiment, once after exposure to each condition.

### Measure of mutual grooming

The participants were asked to complete the Mutual Grooming Scale (version two) (Nelson and Geher, 2007) at the end of the experimental session. The Mutual Grooming Scale is a self-reported 28-item scale (14 items on the frequency of giving grooming and 14 items on the frequency of receiving grooming). The items reflect a wide variety of the forms that grooming takes in humans. The scale measures the frequency of social touches with a “significant other.” Items are scored as follows: 1 (never), 2 (1–6 times per year), 3 (7–12 times per year), 4 (1–3 times per month), 5 (1–3 times per week), 6 (4–6 times per week), or 7 (1 or more times per day). One of the forms proposed by Nelson and Geher (2007) for measuring grooming styles is aggregating the scores from “giving grooming” and “receiving grooming” sub-scales. Herein we adopted this form.

### Task

**Figure 1:**
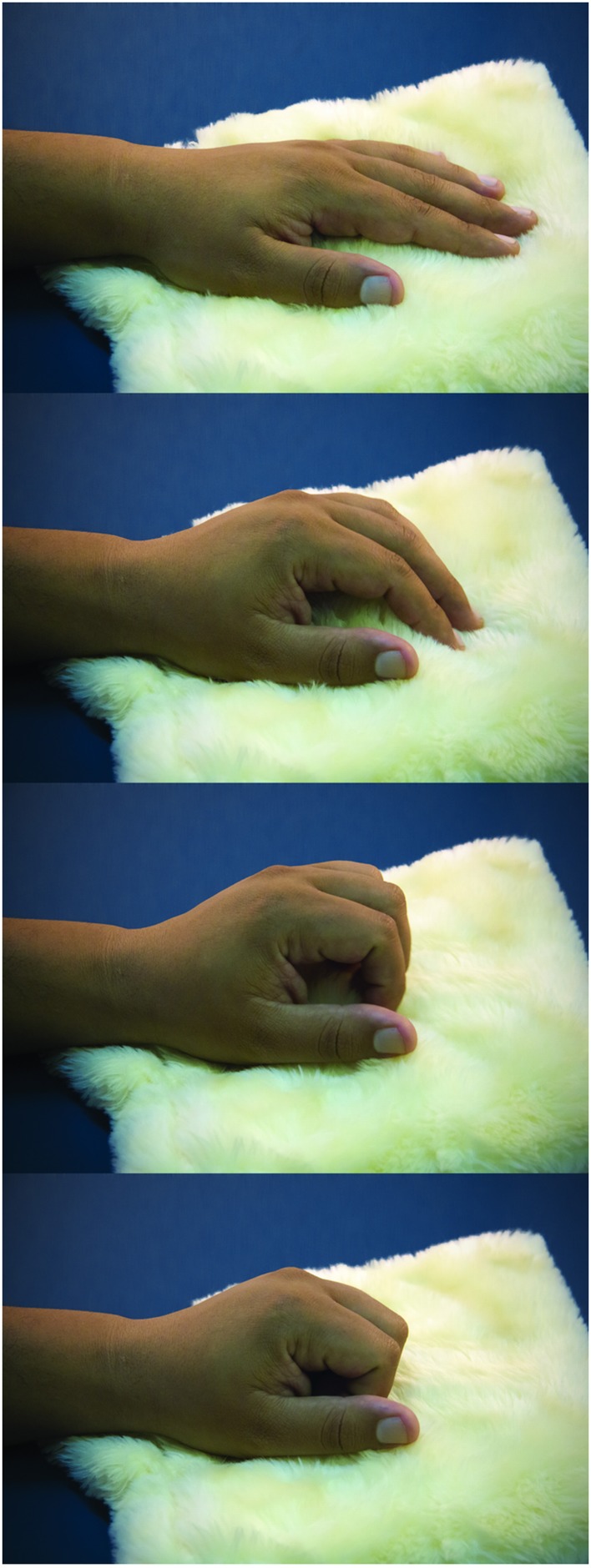
**Task**. The sequential photographs illustrate the paced fingers flexion over the soft cloth, resembling a caress-like movement.

Participants were tested in a sound-attenuated room under dim ambient light. They were asked to sit with both arms comfortably placed over a table. The left wrist and hand rested on a very soft cloth. As Dirnberger et al. (2011) reported the readiness potential to be of higher amplitudes for movements with the non-dominant hand, the task was performed using the non-dominant left hand. The task consisted of a paced single flexion of fingers over the soft cloth (Figure 1). After performing the task, the participant returned the left hand to the resting position.

To compute the readiness potential, paradigms usually employ protocols where participants move in a self-paced rate (with temporal constraints for the rhythm to allow the computation of the readiness potential). Alternatively, as will be described here, participants are asked to perform a voluntary movement at a time of their own choosing, following a trial start cue. Note that this is different from paradigms testing the “contingent negative variation,” an expectancy-related negative wave in anticipation of a mandatory cue (Walter et al., 1964).

### Procedure

Participants rested their head on a forehead/chin supporter (to stabilize EEG recordings) facing a monitor 57 cm ahead. A microcomputer running E-Prime v2.0 software (Psychology Software Tools, Inc.) (Schneider et al., 2002) timed the presentation of the pictures and delivered the triggers. Each trial began with the presentation of a picture on the computer monitor and the delivery of a synchronizing pulse to the electrophysiological recording systems.

A white dot was presented at the center of the screen for the entire session. The height of the pictures on the monitor comprised 15 degrees of visual angle, and the width ranged from 10 to 20 degrees. Picture presentation lasted for 8 s, and inter-picture intervals varied from 10.5 to 11 s. To ensure attention engagement, the participants were instructed to maintain fixation at the central dot and observe each picture carefully. Upon presentation, the participants were instructed to wait for a few seconds and perform the fingers flexion movements with their left hand (Figure 1). This is important to allow the build-up of the readiness potential associated with the self-volition to move the fingers. Herein a training session, with pictures of objects, ensured that the participants waited approximately 5 s to initiate the task. This was accomplished by giving verbal feedback during the training without explicit information about the desired 5-s interval.

The stimuli were presented in a block design; each block consisted of either the 30 “bonding” pictures or the 30 “control” pictures. Each presentation utilized a different randomized sequence within the block. The block with the “bonding” pictures and the block with the “control” pictures were each presented twice during the experimental session (for the second presentation, a newly randomized sequence within the block was applied), resulting in a total of 120 trials. Half of the participants were exposed to the series “bonding”/“control”/“bonding”/“control,” while the others were exposed to the series “control”/“bonding”/“control”/“bonding.” Between the blocks, the participants were instructed to gently free their head from the supporter and complete the list of mood state adjectives or rest for a period. The next block began when the participant resumed his/her position on the supporter. Each participant completed the self-reported mood survey once after the first presentation of the block with the “bonding” pictures and once after the first presentation of the block with the “control” pictures.

At the end of the session, the electrodes were removed, and the participants completed the mutual grooming scale. The total duration of the experiment was approximately 2 h.

### Data analysis

Electromyographic signals of the flexor digitorum superficialis muscle were epoched from 1 s before picture presentation to 10 s after picture presentation and rectified. The 200-ms interval preceding stimulus presentation served as the baseline. For each segment, the onset of movement after picture presentation was attributed to the time of increased EMG activity above basal levels, which was determined by visual inspection. As raised by Hasbroucq et al. (1999) and Van Boxtel et al. (1993), we also observed that this method allows an even more precise detection of the EMG onset than when we applied an automated method. The time intervals between the picture presentation and the onset of movement were averaged separately across the trials for each condition. Average intervals in seconds for the “bonding” and “control” conditions were respectively *M* = 5.0 (*SD* = 0.99) and *M* = 5.1 (*SD* = 0.78); showing that participants implicitly complied with our experimental strategy to allow the recording of the readiness potential.

For each trial, a window of interest covering 2000 ms was set after the onset of movement to estimate the mean amplitude of the EMG for each condition. Before evaluation, we confirmed that peak amplitudes for all participants fell within this window by inspecting mean amplitude values every 500 ms until 3500 ms.

The EEG data were filtered offline using a 30-Hz low-pass digital filter. The readiness potential was described in frontal, central and parietal electrode sites (Shibasaki and Hallett, 2006). Thus, analysis was performed in frontal (Fz, F3, and F4), central (Cz, C3, and C4) and parietal (Pz, P3, and P4) channels. Offline analysis of the data was performed using the EEGLAB version 7.2.9 toolbox (Delorme and Makeig, 2004) with MATLAB 7.0 software (MathWorks, Natick, MA, USA). Eye movement artifacts were removed from the data using the independent component analysis (ICA) tool available in EEGLAB. ICA algorithms are effective for isolating components corresponding to eye blinks (Jung et al., 2000). These components were excluded from data only after visual inspection of the topographic maps demonstrating their proximity to the ocular area and established waveform characteristics.

The negative rise of the readiness potential was described as starting at about 1.5 s before the onset of the muscular activity (Jahanshahi and Hallett, 2003). For each trial, the epoch for EEG analysis was defined as the signal segment from 3000 ms prior to the onset of EMG activity (time zero) until 1000 ms afterwards. The baseline corresponded to the first 500 ms of the epoch, that is, from −3000 to −2500 ms. Epochs in which the EEG signal exceeded ±100 μV were excluded from further analysis. The signal segments for each participant were averaged separately for the trials in the “bonding” condition and the trials in the “control” condition.

A window of interest was set between −1500 and −100 ms to estimate the mean amplitude of the readiness potential. Studies refer to a great inter-individual variability of the *Bereitschaftspotential*, including its absence in some individuals (Dick et al., 1987; Colebatch, 2007). Data from six participants not exhibiting the readiness potential in the “control” and/or “bonding” condition were not further analyzed. Fifteen participants remained for the analysis of readiness potential.

Picture-to-readiness latency was determined by the difference “a–b,” where “a” is the time interval from the picture presentation to the onset of movement (estimated from the EMG recordings), and “b” is the time interval from the onset of readiness to the onset of movement (estimated from the EEG recordings). We estimated the onset of the readiness potential (start of a negative slope) by visual inspection of the averaged signal recorded from channel C4, contralateral to the movement performance. Estimations were undertaken separately for the “bonding” and the “control” conditions. Three experimenters inspected the averaged signals to reach a consensus definition of the onset of readiness.

### Statistical analysis

To assess the differential impact of the stimulus conditions, the scores for the self-reported “hope for closeness” mood after exposure to the “bonding” and to the “control” conditions were compared using the paired two-tailed Student's *t*-test. Similarly, we compared the scores for the self-reported “fear of rejection” mood after exposure to the “bonding” and to the “control” conditions using the paired two-tailed Student's *t*-test.

The amplitudes of the readiness potential were analyzed using repeated measures ANOVA with condition (bonding/control) and channel (F3, Fz, F4, C3, Cz, C4, P3, Pz, and P4) as within-subject factors. To account for data sphericity, we used the Greenhouse-Geisser correction. Fischer *post-hoc* analysis was performed when significant differences were detected.

EMG mean amplitudes were compared between “bonding” and “control” conditions using the paired two-tailed Student's *t*-test.

Picture-to-readiness latencies were compared between “bonding” and “control” conditions using the paired two-tailed Student's *t*-test. Scores on the grooming scale and values of picture-to-readiness latencies were compared using Spearman's correlation, separately for each condition.

In all analyses, *p*-values <0.05 were considered statistically significant.

## Results

### Self-reported mood

Exposure to bonding pictures increased subjective feelings of sociability. The scores for the self-reported “hope for closeness” mood were higher after exposure to the “bonding” condition compared to the “control” condition [*t*_(20)_ = 2.98, *p* = 0.007]. Feelings of isolation decreased; the scores for the self-reported “fear of rejection” mood were lower after exposure to the “bonding” condition compared to the “control” condition [*t*_(20)_ = −2.94, *p* = 0.008]. Whether the dyads in the pictures showed signs of social interaction critically impacted the participants' feelings related to sociability or loneliness.

### Electrophysiology

There was a main effect of channel [*F*_(8, 112)_ = 6.21, *p* < 0.001, ε = 0.46]. *Post-hoc* analysis revealed that the mean amplitude in C4 was significantly higher than the mean amplitudes in F3, Fz, C3, P3, Pz, and P4. Highest amplitude in central contralateral channel C4 (see Table 1) ratifies that the readiness potential is related, as expected, to the movement of the left hand (Shibasaki and Hallett, 2006; Fabiani et al., 2007).

**Table 1:** **Mean amplitudes of readiness potentials (μV) per channel**.

**Channel**	**Mean amplitude**
F3	−2.59
Fz	−2.53
F4	−4.07
C3	−1.72
Cz	−2.89
C4	−4.44
P3	−0.36
Pz	−0.41
P4	−0.95

**Figure 2:**
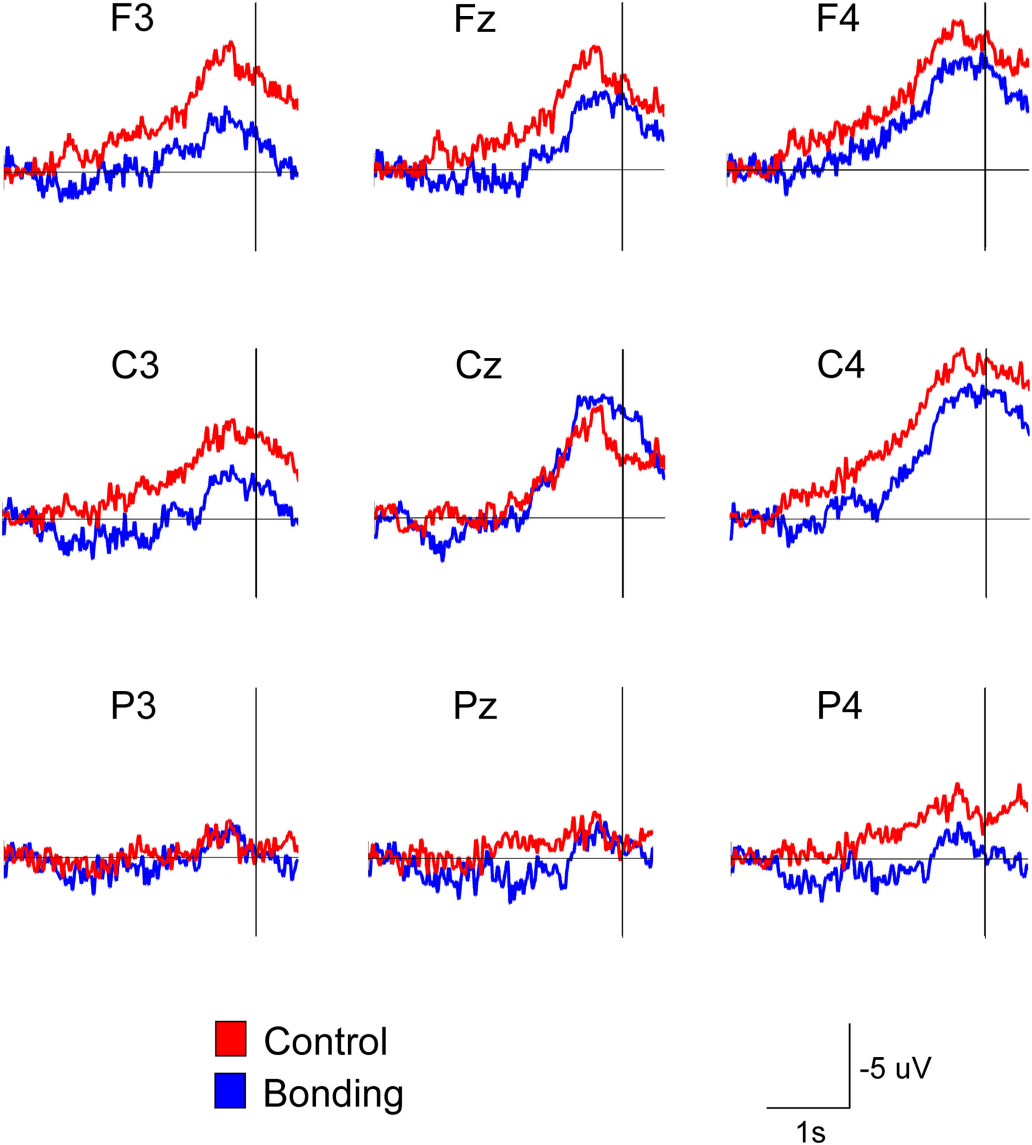
**Attenuation of the readiness potential by previous exposure to bonding pictures**. Grand averages for the nine electrodes in the “bonding” (blue) and “control” (red) conditions are depicted. Vertical lines represent movement onset.

Most importantly, there was a main effect of condition [*F*_(1, 14)_ = 11.86, *p* = 0.004, η^2^_*p*_ = 0.459, power = 0.892]. Performing the caressing movement under the “bonding” condition resulted in lower mean amplitudes of readiness potential than were observed under the “control” condition. There was no significant interaction between condition and channel (*p* = 0.114). Figure 2 presents the grand averages of the readiness potentials in the “bonding” and “control” conditions for all channels. Table 2 depicts the mean amplitudes values of readiness potentials in each condition averaged across participants.

**Table 2:** **Mean amplitudes of readiness potentials (μV) for “Bonding” and “Control” conditions**.

	**Frontal channels**			**Central channels**			**Parietal channels**		
	**F3**	**Fz**	**F4**	**C3**	**Cz**	**C4**	**P3**	**Pz**	**P4**
Control	−3.85	−3.58	−4.85	−2.88	−2.77	−5.46	−0.51	−1.03	−1.98
Bonding	−1.34	−1.49	−3.29	−0.55	−3.02	−3.42	−0.20	0.21	0.08

**Figure 3:**
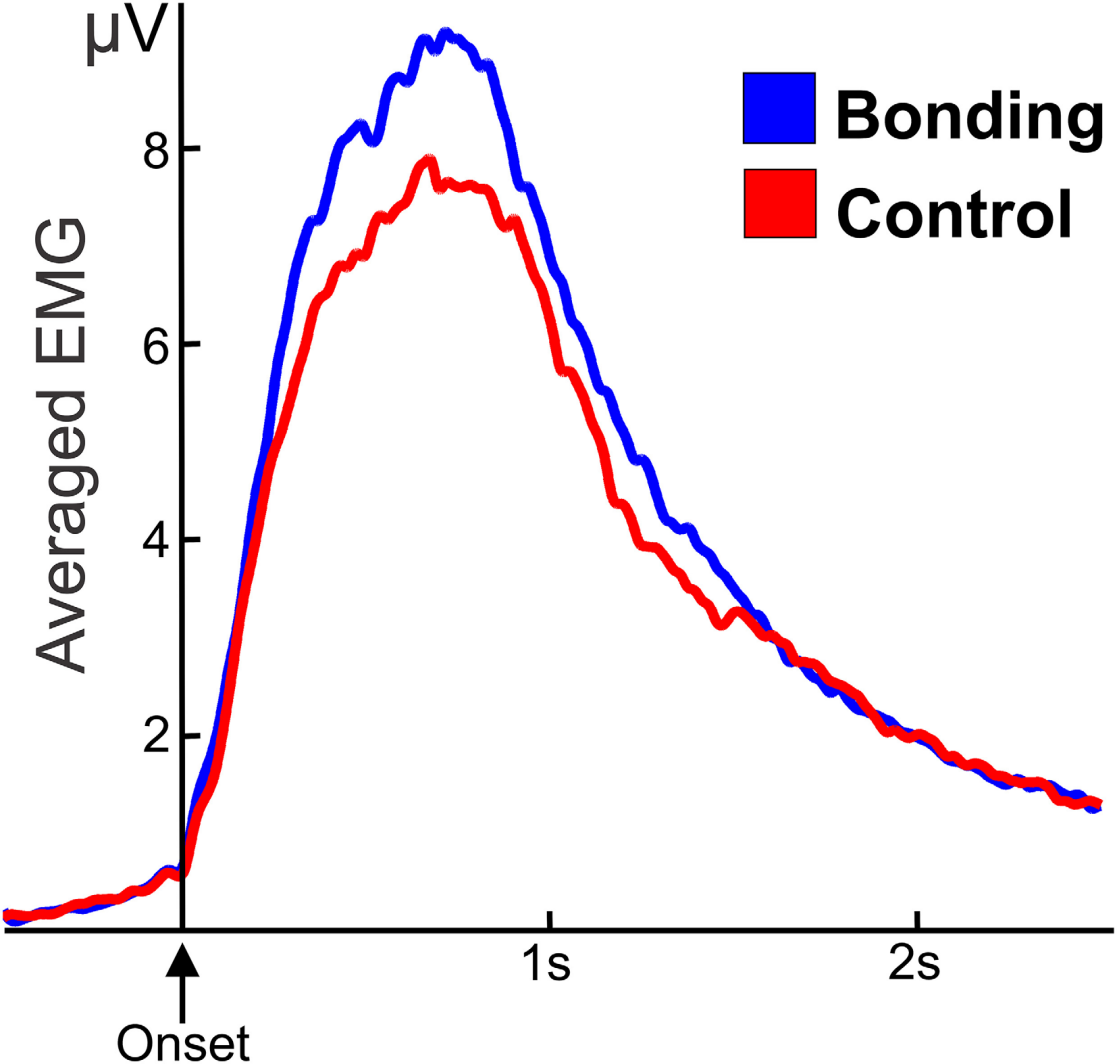
**Averaged and smoothed electromyographic activity (potentials in μV) of fingers flexor during the caressing of the soft cloth**. In the “bonding” (blue) condition activity is higher than in the “control” (red) condition. Zero in the abscissa represents movement onset.

The mean amplitude of the electromyographic activity of fingers flexor performing the caress movement was significantly higher for the “bonding” condition compared to the “control” condition [*t*_(20)_ = 2.25, *p* = 0.036]. Averaged electromyographic activity from the participants in the two conditions is shown in Figure 3.

The rising of the negative slope of the readiness potential took place on average *M* = 4.08 s (*SD* = 1.02) and *M* = 4.05 s (*SD* = 0.80) after pictures' onset, respectively for the bonding and control conditions. Interestingly, although movement preparation was self-volitional, grooming styles modulated the latencies to initiate the readiness potentials. Habitual “groomers” initiated the motor preparation earlier than infrequent groomers, so that latencies correlated negatively with grooming scores in either conditions (“bonding”: rho = −0.63, *p* = 0.011; “control”: rho = −0.77, *p* = 0.001).

## Discussion

We employed exposure to bonding cues and electrophysiological (brain and muscles) recordings related to a task consisting of a caress-like movement. The choice of paradigm relied on (i) the human capacity to perceive subtle cues of social interactions (Over and Carpenter, 2009; Centelles et al., 2011), and (ii) the recognized predisposition to caress: human beings enjoy body nearness and contact and are especially motivated toward caressing each other (Carter et al., 2005; Guest et al., 2009; Morrison et al., 2010). Exposure to the bonding pictures compared to the control pictures increased subjective feelings of sociability and decreased feelings of isolation. Caressing the soft cloth seemed related to mutual grooming, since individual variability in grooming styles modulated the latencies to initiate the readiness potentials. Taken together, the experimental paradigm was well adapted to test for the presence of pre-set neural circuits associated with caress-like actions. As hypothesized, under the exposure to bonding pictures the readiness potential preceding caressing of the soft cloth showed lower amplitudes, and the muscles enrolled in the caress movement showed higher electromyographic activity, compared to performing the same movement under exposure to control pictures.

It is assumed that social bonds have consequences for virtually all aspects of behavior and that the motivational and behavioral systems underlying them are evolutionarily ancient traits (Carter et al., 2005; Decety et al., 2012). The perception of actual or potential psychological distance from close others is alarming, distressing, and painful (Eisenberger and Lieberman, 2004; Kross et al., 2011). Automatic motivation to seek companionship provides, among other roles, an effective protection against predation; being alone is a condition that stimulates fear, while being with a companion is highly rewarding and greatly reduces fear (Bowlby, 1973). As many studies have shown, social bonding is not only rewarding but also necessary for our health and well-being (Feldman et al., 2010; Field, 2010; Cacioppo and Cacioppo, 2012; Decety and Svetlova, 2012). Epidemiological studies suggest that the presence of social bonds are important predictors of speed of recovery and subsequent longevity following illnesses (Carter et al., 2005). Also, Luo et al. (2012), among others, indicated that loneliness is a risk factor for morbidity and mortality. Further, there is evidence in humans that impairments of affiliative behaviors are associated with maladaptive interpersonal patterns and psychiatric disorders (Bora et al., 2009).

Social touch is very important for non-verbal communication, environmental adaptability, good health and attachment bonds (for a review, see Gallace and Spence, 2010). Allo-grooming (the grooming of others), which is considered a special kind of social touch, assumes in primates (including humans) a particularly important and prominent role in social bonding (Dunbar and Shultz, 2007; Dunbar, 2010). Although mutual grooming in humans has received much less attention from researchers than in other primates, the assessment of mutual grooming in human dyadic relationships through a scale revealed that allo-grooming is quite frequent in humans (Nelson and Geher, 2007). Indeed, caressing another person's skin is fairly pleasant for the toucher (Guest et al., 2009; Morrison et al., 2010). Actively touching something soft seems to be comforting since infancy. Classic studies by Harlow (e.g., Harlow, 1958) showed that infant monkeys sought to touch and cling on a surrogate “mother” covered with soft cloth in clear preference to a wire-framed surrogate. Contact comfort with the soft surrogate was preferred even when the wire surrogate was the one that provided nutrition. In adult monkeys, giving grooming is stress-reducing and this effect can be even stronger than receiving grooming (Shutt et al., 2007).

It has been proposed that viewing pictures of newborns elicits a phylogenetically based readiness for response preparation which would involve protect, embrace, hold close and groom the baby (Brosch et al., 2007). A recent functional neuroimaging study showed that activity in the premotor cortex was modulated by passive exposure to pictures of infant faces compared to adult faces (Caria et al., 2012). The authors speculated that viewing infant faces might prompt cortical motor predispositions to interact with them. Our study allowed a deeper insight and a workable test of existing motor predispositions toward social interaction and physical contact. We induced affiliation mood with pictures of infant-infant or adult-infant interacting dyads. It is worth noting that in our study, infants were equally present in both the “bonding” and the “control” conditions, so that the social interaction is, *per se*, salient enough to modulate the excitability of circuits associated with a “compatible” action, that is, caress-like movement.

Direct cortical micro-stimulation studies in monkeys (Graziano, 2006) and humans (Desmurget et al., 2014) revealed the representation of motor repertoires for ethologically relevant actions. In monkeys, Graziano (2006) observed that some stimulation sites evoked hand movements possibly associated with allo-grooming. Our data bring support to the cortical representation of caressing movements in humans. The contrast between the compatible “bonding” condition and the “control” condition showed that the former significantly affected the motor planning in the brain (reduced readiness potential) and the movement execution of caressing the soft cloth (increased fingers flexor electromyography). Many other cortical and subcortical regions, for example the several nuclei of the basal ganglia (Hikosaka et al., 2014), are implicated in intricate inhibitory and disinhibitory mechanisms that enable a subject to perform value-laden actions. The present work was not aimed to tackle intrinsic mechanisms of neural circuits, and hypotheses on the neural basis underlying the results are speculative at most. Our conjecture is that exposure to bonding scenes pre-activates the circuits for caressing movements which would turn them into a “ready-to-go” state. In contrast, no such pre-activation occurs in the absence of bonding cues. When the action planning starts to build up triggered by the participant's decision to move, there will be a facilitation for the compatible condition. Activation of motor circuits by cues compatible with a given movement has already been shown in the monkey pre-motor cortex. Kohler et al. (2002) recorded from such neurons that discharged when the animal performed a specific action, and also when the monkey was exposed to compatible visual and/or auditory stimuli (e.g., neurons that discharged similarly when the monkey broke a peanut as well as when it heard a peanut being broken).

Social touch is a very important component of social bonding. Previous neurophysiological studies of social touch have emphasized the perspective of the touch's recipient (e.g., Löken et al., 2009; Gordon et al., 2013). The present work contributed data from the perspective of basic implicit predispositions to act toward social engagement through hand movements resembling caressing.

## Author contributions

Rafaela R. Campagnoli, Leticia Oliveira, Mirtes G. Pereira, Isabel A. David, Claudia D. Vargas and Eliane Volchan designed research; Rafaela R. Campagnoli, Laura Krutman and Isabela Lobo performed research; Rafaela R. Campagnoli, José M. Oliveira, Isabel A. David, Laura Krutman and Eliane Volchan analyzed data; Rafaela R. Campagnoli, Isabel A. David, José M. Oliveira and Eliane Volchan wrote the paper.

### Conflict of interest statement

The authors declare that the research was conducted in the absence of any commercial or financial relationships that could be construed as a potential conflict of interest.
