# Supplementary material for: Preparing to caress: a neural signature of social bonding
Source: Front Psychol. 2015 Jan 28;6:16. doi: 10.3389/fpsyg.2015.00016 (PMC4309179; doi:10.3389/fpsyg.2015.00016)
Supplement: Supplementary file 1 [file Image1.PDF]

Supplementary Material

Bonding | Control

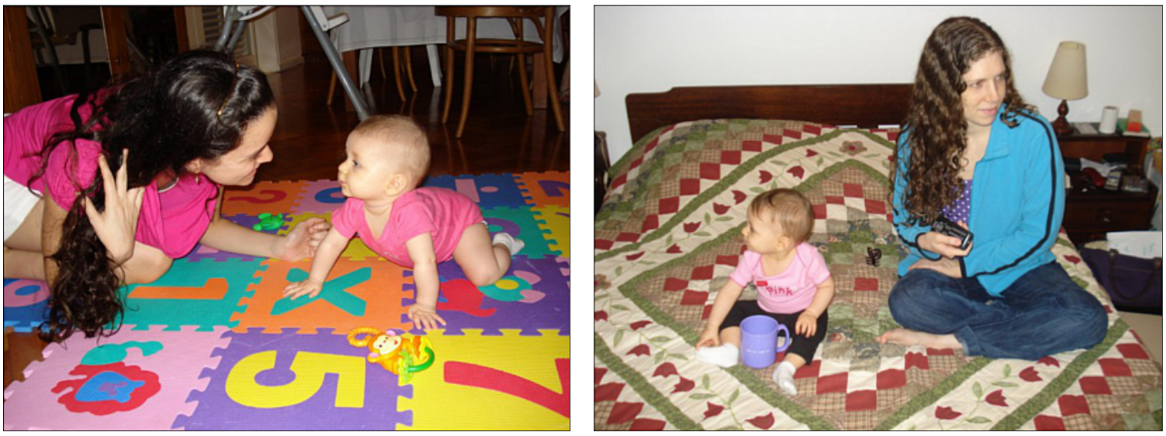

Caption:
Two examples of pictures, one for each condition. The “bonding” condition (left) shows a scene of an interacting dyad. The “control” condition (right) shows a similar scene, but with a non-interacting dyad.
